# Indication and eligibility of glioma patients for awake surgery: A scoping review by a multidisciplinary perspective

**DOI:** 10.3389/fonc.2022.951246

**Published:** 2022-09-21

**Authors:** Giorgio Fiore, Giorgia Abete-Fornara, Arianna Forgione, Leonardo Tariciotti, Mauro Pluderi, Stefano Borsa, Cristina Bana, Filippo Cogiamanian, Maurizio Vergari, Valeria Conte, Manuela Caroli, Marco Locatelli, Giulio Andrea Bertani

**Affiliations:** ^1^ Department of Neurosurgery, Fondazione Istituto di Ricovero e Cura a Carattere Scientifico (IRCCS) Ca' Granda Ospedale Maggiore Policlinico, Milan, Italy; ^2^ Department of Pathophysiology and Transplantation, University of Milan, Milan, Italy; ^3^ Department of Neuropathophysiology, Fondazione Istituto di Ricovero e Cura a Carattere Scientifico (IRCCS) Ca' Granda Ospedale Maggiore Policlinico, Milan, Italy; ^4^ Neuro Intensive Care Unit, Fondazione Istituto di Ricovero e Cura a Carattere Scientifico (IRCCS) Ca' Granda Ospedale Maggiore Policlinico, Milan, Italy

**Keywords:** awake, awake surgery, eligibility, indication, gliomas, brain tumors, brain mapping, exclusion criteria

## Abstract

**Background:**

Awake surgery (AS) permits intraoperative mapping of cognitive and motor functions, allowing neurosurgeons to tailor the resection according to patient functional boundaries thus preserving long-term patient integrity and maximizing extent of resection. Given the increased risks of the awake scenario, the growing importance of AS in surgical practice favored the debate about patient selection concerning both indication and eligibility criteria. Nonetheless, a systematic investigation is lacking in the literature.

**Objective:**

To provide a scoping review of the literature concerning indication and eligibility criteria for AS in patients with gliomas to answer the questions:1) "What are the functions mostly tested during AS protocols?" and 2) "When and why should a patient be excluded from AS?".

**Materials and methods:**

Pertinent studies were retrieved from PubMed, PsycArticles and Cochrane Central Register of Controlled Trials (CENTRAL), published until April 2021 according to the PRISMA Statement Extension for Scoping Reviews. The retrieved abstracts were checked for the following features being clearly stated: 1) the population described as being composed of glioma(LGG or HGG) patients; 2) the paper had to declare which cognitive or sensorimotor function was tested, or 2bis)the decisional process of inclusion/exclusion for AS had to be described from at least one of the following perspectives: neurosurgical, neurophysiological, anesthesiologic and psychological/neuropsychological.

**Results:**

One hundred and seventy-eight studies stated the functions being tested on 8004 patients. Language is the main indication for AS, even if tasks and stimulation techniques changed over the years. It is followed by monitoring of sensorimotor and visuospatial pathways. This review demonstrated an increasing interest in addressing other superior cognitive functions, such as executive functions and emotions. Forty-five studies on 2645 glioma patients stated the inclusion/exclusion criteria for AS eligibility. Inability to cooperate due to psychological disorder(i.e. anxiety),severe language deficits and other medical conditions(i.e.cardiovascular diseases, obesity, etc.)are widely reported as exclusion criteria for AS. However, a very few papers gave scale exact cut-off. Likewise, age and tumor histology are not standardized parameters for patient selection.

**Conclusion:**

Given the broad spectrum of functions that might be safely and effectively monitored *via* AS, neurosurgeons and their teams should tailor intraoperative testing on patient needs and background as well as on tumor location and features. Whenever the aforementioned exclusion criteria are not fulfilled, AS should be strongly considered for glioma patients.

## 1. Introduction

In the 1950s the neurosurgeon Wilder Penfield firstly proposed an innovative technique to surgically treat epilepsy, without total anesthesia, but only through local and intermittent sedation and analgesia in order to identify the epileptogenic focus and the sensorimotor area by direct electrical stimulation of cerebral cortex (1, 2). In the past decades, this technique has grown importance and, nowadays, awake surgery (AS) represents a key treatment not only for epilepsy (3, 4), but also for resection of brain neoplastic intra-axial lesions, mainly gliomas (5–7), and deep brain stimulation in patients affected by Parkinson disease (8, 9). The central aspect of this technique is that the patient is conscious and actively participates in neuropsychological testing during surgical operations, helping neurosurgeons to avoid permanent brain function impairment. Different anesthesiologic and sedation strategies were described, with the two principal techniques being represented by the asleep-awake-asleep (AAA) and the fully awake craniotomy (FA) (10). In the former, the patient is initially under general anesthesia and is awakened after completing the craniotomy, when the surgeon is ready to start the intraoperative mapping of cognitive and motor pathways. In the FA procedure, the patient is awake during the entire procedure; consciousness and pain are controlled by local anesthetics and sedation as needed.

In the specific case of AS used for resection of gliomas, this technique aims at minimizing the risks for postoperative permanent neurological and cognitive deficits while maximizing the extent of resection (5, 11). It is now recognized that postoperative permanent impairment of sensorimotor and cognitive functions, such as aphasia, apraxia, visuospatial deficits, and other dysexecutive syndromes, is characterized by neurological, functional, and behavioral symptoms that contribute to loss of autonomy and undermine the patients' quality of life (QoL) (12). The importance of a safe procedure for the resection of gliomas infiltrating eloquent areas is well described in the literature and several techniques of intraoperative cortical and subcortical mapping have been proposed (5, 6, 13, 14). In this view, AS permits intraoperative cortical and subcortical mapping of cognitive functions and the continuous assessment of motor responses, allowing neurosurgeons to stop the resection according to patient functional boundaries thus preserving long-term patient integrity (13). Maximizing extent of resection (EOR) is particularly important when considering patient survival and prognosis: several studies demonstrated that gross total resection (GTR) and, when feasible, supratotal resection, can improve progression-free survival (PFS) and overall survival (OS) both in lower-grade gliomas (LGG) and high-grade gliomas (HGG) (15–19).

Given the patient discomfort and the increased risks of the awake setting, namely intraoperative seizures, difficult airway management and brain swelling/bleeding, the growing importance of AS in surgical practice promotes the debate about patient selection concerning both indication and eligibility criteria. Due to special neurosurgical, anesthesiologic, neuropsychological and neurophysiological issues, eligibility needs to be verified by different specialists in a multidisciplinary setting involving neurosurgeons, neurophysiologists, anesthesiologists, neuropsychologists and linguists. The literature is lacking about specified and coherent criteria from all these perspectives that often depend on each institution's practice and surgical team expertise, thus failing in reporting specific and well-established indications for AS procedure.

This scoping review aims to systematically investigate the literature concerning indication and eligibility criteria for AS in glioma patients to answer the questions: 1) "What are the functions mostly tested during AS protocols?" and 2) "When and why should a patient be excluded from AS?". To our knowledge, this is the first scoping review addressing the selection process concerning the indication and eligibility of patient candidates for AS.

## 2. Materials and methods

### 2.1 Search strategy

Pertinent studies published until April 2021 were retrieved from PubMed, PsycArticles and Cochrane Central Register of Controlled Trials (CENTRAL), according to the Preferred Reporting Items for Systematic Reviews and Meta-Analyses Statement Extension for Scoping Reviews (PRISMA-ScR) (20). Grey literature and unpublished papers were not included. To search for appropriate papers, we entered the following keys in the "Title" or "Abstract" fields: ["Awake Surgery" AND (indications OR selection)], ["Awake Surgery" AND (Glioma OR "brain tumors")], ["Awake craniotomy" AND (Glioma OR "brain tumors")], [(Glioma) AND (intraoperative monitoring)] and [intraoperative brain mapping AND (Glioma OR "brain tumors")]. Additional research was conducted through the screening of the reference lists reported in the included papers. Among all the retrieved records, we excluded duplicates and papers that did not fit the conceptual framework of the study; we did not include, for example, studies addressing pathologies other than gliomas (either LGG or HGG) or which did not adopt an awake protocol. The kinds of study design eligible for this review were observational prospective and retrospective case series, case-control studies, cohort studies and randomized-controlled trials (RCT). We excluded articles in other languages than English.

### 2.2 Study selection

The authors conducted the first examination on the retrieved abstracts, including the researches that had the following features clearly stated: 1) the population described as being composed of glioma (LGG or HGG) patients, independently of age; 2) the paper had to declare which cognitive or sensorimotor function was tested during the awake phase.

The second screening of the retrieved abstracts was conducted to identify the inclusion/exclusion criteria for AS. During this procedure, the first inclusion criterion was maintained and a different second one was: 2 *bis*) the decisional process of inclusion/exclusion for AS had to be described from at least one of the following perspectives: neurosurgical, neurophysiological, anesthesiologic and psychological/neuropsychological.

Two different tables including the main variables of interest were charted for both selection processes. The former comprised: the testing of language, sensorimotor and visuospatial pathways, or other superior cognitive functions, such as executive functions or emotions, and the hemisphere being tested; when available, the intraoperative tests being employed were reported. The second table encompassed the following variables: neurosurgical, neurophysiological, anesthesiologic, psychological/neuropsychological or other exclusion criteria.

### 2.3 Statistical analysis

All statistical analyses, pie charts and histograms were performed using IBM SPSS version 25.0, International Business Machines Corp, New York, USA.

Well diagrams were plotted using BioVinci version 2.0, BioTuring, San Diego, USA.

## 3. Results

### 3.1 Study selection

Overall, 1.182 references were retrieved using the aforementioned keywords. Two authors (G.A-F. and A.F.) independently examined the titles and abstracts of the studies. After removing duplicates, a total of 911 references were identified for the title and abstract screening. The full text of 418 studies was obtained and assessed. In case of selection discrepancy, a third author (G.F.) assessed the article. A total of 189 studies, including 8956 patients, matched the inclusion criteria of this scoping review. Particularly:

- The first selection procedure included 178 studies published from 1994 to 2021. On this wise, data from tested functions of 8004 patients were included in this review. All the studies reported surgical case series of which 42 (24%) included prospective analyses. The results of the first selection procedure are reported below as Indication to AS: Tested Functions.

- The second screening encompassed 45 studies that were conducted on 2645 glioma patients and stated the inclusion/exclusion criteria being adopted for AS eligibility. The studies were published from 2004 to 2021. All the studies reported surgical case series of which 18 (40%) included prospective analyses. The results of this selection procedure are reported below as Eligibility criteria for AS: Exclusion Criteria.

**Figure 1:**
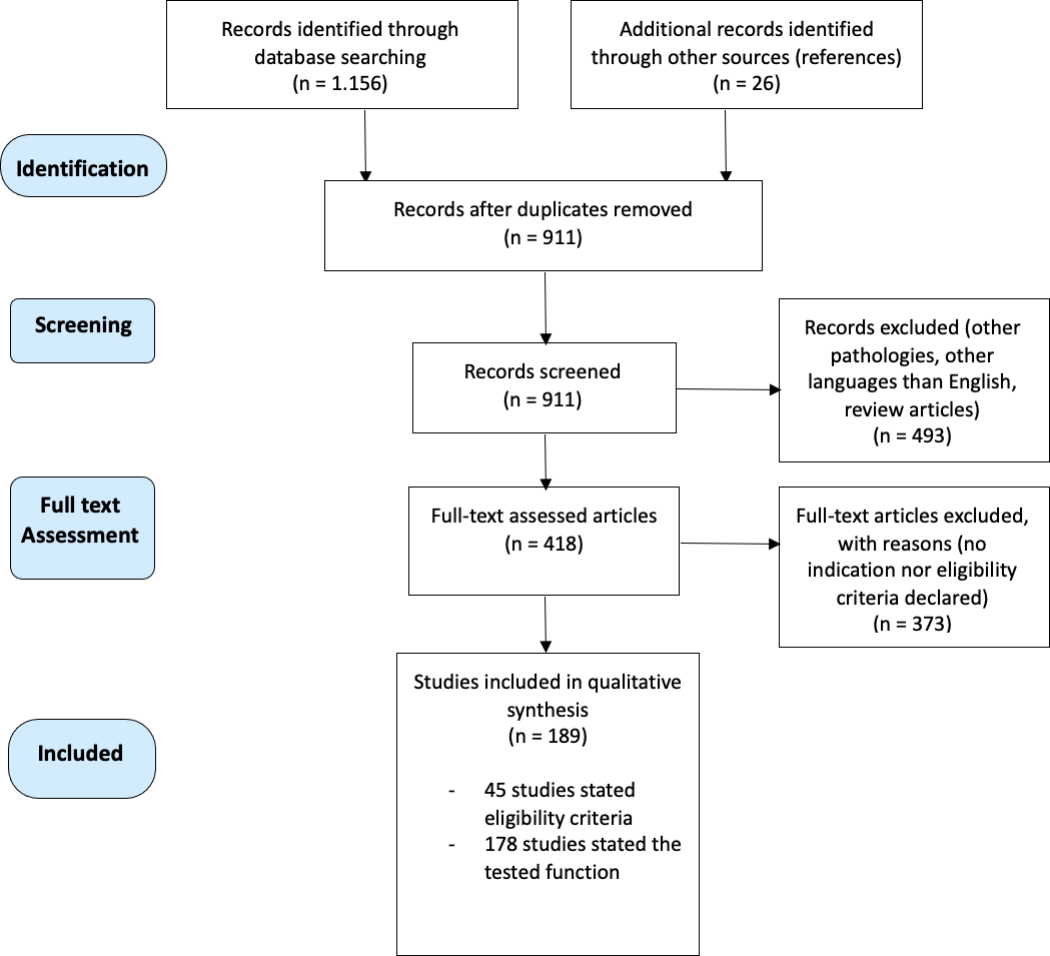
Flowchart of the selection process of this scoping review, according to PRISMA Statement Extension for Scoping Reviews (PRISMA-ScR).

The process of article selection is reported in **Figure 1**.

### 3.2 Indication to AS: Tested functions

**Figure 2:**
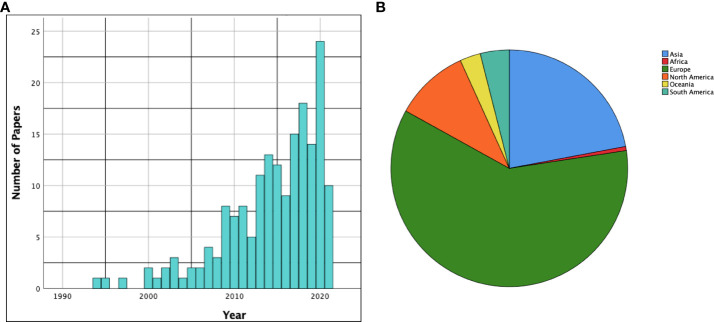
**(A)** The histogram shows the time distribution (by years) of the papers included in the scoping review. **(B)** The pie chart summarizes distribution of the included papers among the various continents.

One hundred and seventy-eight studies described the main function/functions being tested during awake surgery. As deducible from **Figure 2**, an increasing number of papers in the literature commenced focusing on the functions being tested during AS starting from the 2000s.

One hundred and seven studies (60%) were conducted in Europe, while thirty-nine (22%) were conducted in Asia. North America was the third most represented continent with eighteen articles (10%). A summarizing pie chart is available in **Figure 2**.

The main indication for AS was the monitoring of language spectrum functions which was addressed in 149 (84%) articles. The object naming task was the principal test administered during AS being employed in 104 (58%) studies. The verb naming task was utilized in 21 (12%) studies. Semantic association, comprehension, repetition, reading and writing was respectively addressed in 21 (12%), 9 (5%), 11 (6%), 28 (16%) and 2 (1%) papers. Three major studies focused on multilanguage patients and the use of a translator in the theatre during AS (21–23).

The second most frequent indication for AS was monitoring of the sensorimotor pathway which was assessed in 84 (48%) surgical series. The evaluation of fine movements and praxis were addressed in overall 22 (12%) studies.

The visuospatial pathway, including spatial awareness, was the third most assessed function being tested in 27 (15%) studies, with the line bisection test as the usual implemented task.

Seventeen (10%) papers focused on executive functions, such as inhibition and working memory, that were frequently assessed by the Stroop test and the double-task test.

Eight (5%) studies employed a test to identify emotional pathways and areas related to the theory of the mind.

**Figure 3:**
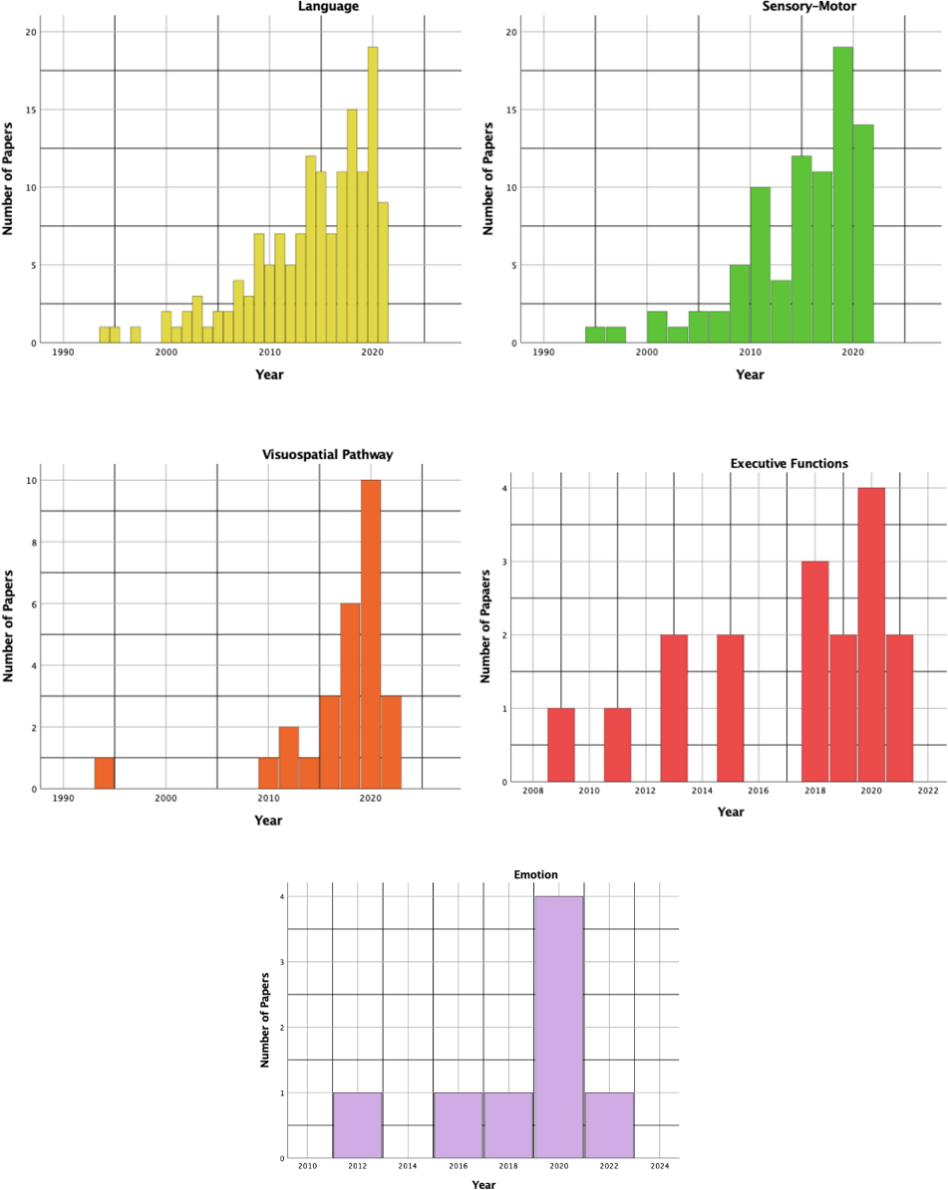
The histograms show the time distribution (by years) for each function being tested in the included studies.

**Figure 4:**
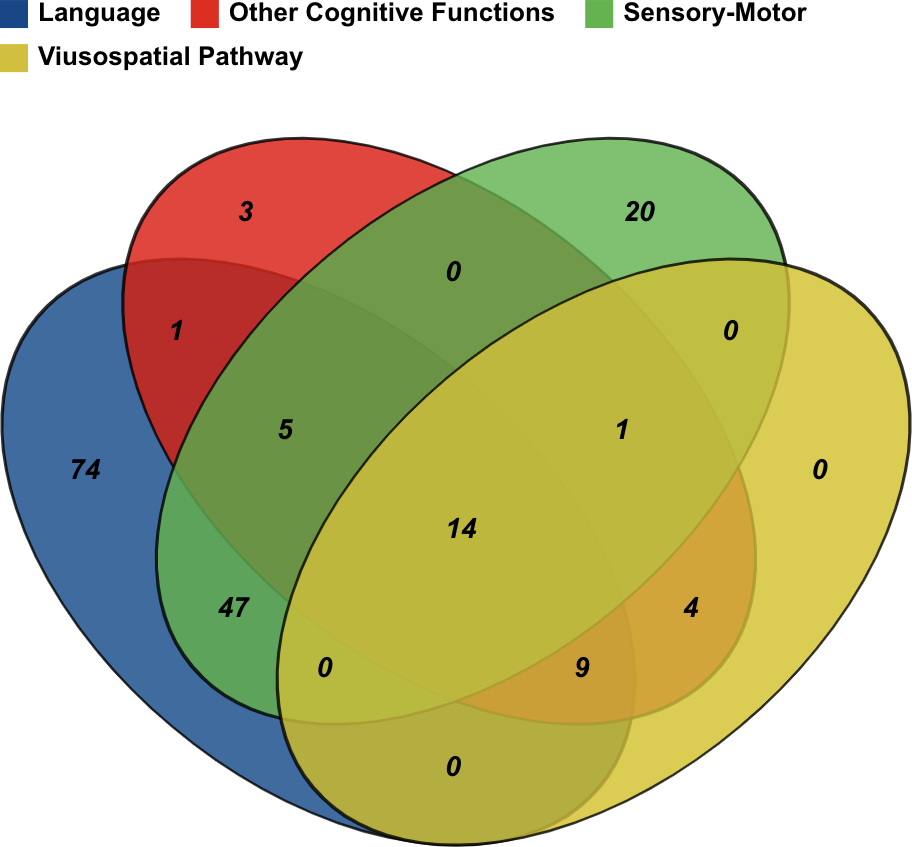
The Well diagram shows the tested functions in the included surgical case series.

**Figure 3** pointed out as all the aforementioned functions have been objects of increasing interest in the literature over years, even if testing of executive function, emotional and visuospatial pathways has grown mainly in the last decade. The Well diagram showing the functions being tested in the surgical case series included in this review is plotted in **Figure 4**.

### 3.3 Eligibility criteria for AS: Exclusion criteria

Patients age ranged from 9 to 90 years. Particularly, 27 studies involved only adult populations and 9 both adults and children/adolescents (< 18 years). Two studies included only pediatric populations.

Regarding the anesthesiologic protocol, 21 studies reported patients as being operated on with AAA procedures (46,7%), whilst 22 articles adopted fully awake craniotomy (FA) protocols (48,9%). One paper embedded both the procedures (2,2%).

Among the articles which reported glioma histological grade, we found a total of 1129 patients affected by LGGs and 1138 HGGs.

The main features of these studies with the relative inclusion/exclusion criteria for AS are reported in **Table 1**.

**Table 1:** Main characteristics of the studies, including relative inclusion and exclusion criteria.

Authors and year	Number of patients	LGG vs HGG	Kindof Anesthesia	Psychological	Neuropsychological	Neurological and neurosurgical	Aenesthesiological	Other conditions
Norrelgen F, et al., 2020 (24)	27	17 vs 10	AAA		Severe Aphasia,	Previous Surgery		
Li, T., et al., 2015 (25)	91	66 vs 25	AAA	Disorientation	Cognitive deficit	Neurological deficits		Age: not under 16 years old; not over 60 years old.
Alimohamadi M., et al., 2016 (26)	10	7 vs 3	FA	Psychopatology	Severe Aphasia, Cognitive deficits	Previous Surgery	Obesity, Cardiovascular disease, difficult airways	Age: over 75 years old
Saito T., et al., 2016 (27)	82	46 vs 36	FA			Previous Surgery		
Krieg SM., et al., 2013 (28)	8	4 vs 4	AAA		Severe Aphasia, Cognitive deficits	AS only for lesions infiltrating left pre or post-central gyrus.		
Kuribara T., et al., 2020 (29)	136	54 vs 82	AAA		Severe Aphasia			
Benyaich Z., et al., 2020 (30)	20	18 vs 2	AAA	Inability to cooperate		KPS of < 70	ASA ≥ 3	Age: over 10 years old
Nossek E., et al., 2013 (31)	424	80 vs 283	FA	Inability to cooperate, anxiety	Severe dysphasia			
Bertani G., et al., 2009 (6)	275	275 LGG	AAA	Inability to cooperate			Obesity, Cardiovascular disease, difficult airways	Age: over 65 years old
Huguet L., et al., 2020 (32)	17	17 vs 0	AAA	Inability to cooperate, lack of motivation, alexithymia, recurrent depression, OCD		Outside eloquent areas		
Manchella S., et al., 2011 (33)	16	5 vs 11	AAA		Severe dysphasia	Outside eloquent areas		
Nabavi A., et al., 2008 (34)	34	NA	FA	Inability to cooperate	Severe Aphasia (< 50% language tests), score MMSE < 23	Neurological deficits, Outside eloquent areas		
Zigiotto L., et al., 2020 (35)	16	NA	AAA	Inability to cooperate, anxiety, confusion	Severe Aphasia	Neurological deficits		
Balogun JA., et al., 2019 (36)	1	16 HGG	AAA			KPS of < 70	Obesity, Cardiovascular disease, difficult airways, respiratory diseases	
Pichierri A., et al., 2019 (37)	20	11 vs 9	AC e AAA	Inability to cooperate, anxiety, phobias, altered mental status	Severe dysphasia	Outside eloquent areas	Obesity (BMI > 35), Cardiovascular disease, obstructive sleep apnea, difficult intubation, active acute or chronic cough, uncontrolled seizures	
Hulou MM., et al., 2015 (38)	25	9 vs 16	FA	Inability to cooperate, emotional instability, developmental delay	Severe Aphasia			
Giussani C., et al., 2011 (39)	35	10 vs 25			Severe Aphasia			
Bello L., et al., 2007 (13)	88	44 vs 44	AAA		Severe Aphasia			
Groshev A., et al., 2017 (40)	44	4 vs 40	FA	Anxiety		Severe intracranial hypertension	Obstructive sleep apnea, history of postanaesthesia nausea and vomiting, bleeding diasthesis, seizures	
Maldaun MV., et al., 2014 (41)	42	14 vs 28	AAA			Outside eloquent areas		
Lu J., et al., 2013 (42)	30	19 vs 11	FA		Severe Aphasia, score MMSE < 23	Outside eloquent areas, severe intracranial hypertension	Pacemaker, obstructive sleep apnea	Not pediatric population
Chacko AG., et al., 2013 (43)	67	36 vs 31	AAA	Inability to cooperate		Outside eloquent areas		Age: not under 13 years old
Klijn E., et al., 2013 (44)	8	8 vs 0	AAA			Outside eloquent areas, suspected LGG		
Leuthardt EC., et al., 2011 (45)	12	4 vs 8	FA				Obesity, difficult airways, severe diabetes mellitus	
Sarubbo S., et al., 2011 (46)	12	12 LGG	AAA	Inability to cooperate, psychiatric disorders	Severe Aphasia (error rate higher than 25% at naming tests)			
Goebel S., et al., 2010 (47)	25	13 vs 12	FA	Anxiety, depression, severe psychological distress	Severe Aphasia, score MMSE < 23, neglect, impairments in concentration, sustained attention, severely disinhibited, apathic, or disorganized behavior.	Outside eloquent areas		
Sanai N., et al., 2008 (48)	250	124 vs 126	FA	Emotional instability, confusion, decreased level of consciousness	Severe Aphasia, Severe dysphasia	Outside eloquent areas		
Picht T., et al., 2006 (49)	20	20 LGG	FA	Inability to cooperate	Severe Aphasia		Obstructive sleep apnea, uncontrolled seizures	
Klimek M., et al., 2004 (50)	1	1 HGG	FA	Inability to cooperate, agitation, restlessness				
Kwinta, B.M., et al., 2021 (51)	25	17 vs 8	AAA	Inability to cooperate		Lovett Scale < 3, Outside eloquent areas		
Coskun, E., 2020 (52)	109	17 vs 92	FA	Depression, psychotic disorders, claustrophobia	Severe Aphasia, Cognitive deficits			
Hejrati, N., et al., 2019 (53)	14	5 vs 9	FA	Inability to cooperate	Severe Aphasia		Obesity, severe gastroesophageal reflux	
Wang, Y.-C., et al., 2019 (54)	41	19 vs 22	AAA	Inability to cooperate	Severe dysphasia			
Sollmann, N., et al., 2018 (55)	60	14 vs 46	AAA		Severe Aphasia			Age: not under 18 years old
Leal, R.T.M., et al., 2018 (56)	13	5 vs 8	FA	Untreated psychiatric condition, claustrophobia	Cognitive deficits	Outside eloquent areas		Patient refusal
Sitnikov, A.R., et al., 2018 (57)	54	35 vs 19	AAA	Psychoemotional lability	Severe Aphasia	Hemiplegia, Outside eloquent areas		
Leal, R.T.M., et al., 2017 (58)	11	4 vs 7	FA	Untreated psychiatric condition, claustrophobia	> 20 % of errors at preoperative object naming test	MRCMS < 2, Outside eloquent areas		
Bunyaratavej, K., et al., 2016 (59)	27	16 vs 11	FA	Inability to cooperate	> 20 % of errors at preoperative object naming test	Lovett Scale < 3		
Krieg, S.M., et al., 2014 (60)	30	8 vs 22	FA		Severe Aphasia		Pacemaker	Age: not under 18 years old
Garavaglia, M.M., et al., 2014 (61)	10	NA	FA	Inability to cooperate, panic attacks		Neurological deficits, large sizes with midline shift	Obesity, obstructive sleep apnea, difficult airways, severe chronic obstructive pulmonary disease/asthma, severe gastroesophageal reflux	
Deras, P., et al., 2012 (62)	140	NA	AAA				Severe asthma, severe reduction of mouth opening, partial airway obstruction, severe gastroesophageal reflux	
Santini, B., et al., 2012 (63)	21	8 vs 13	FA	Psychiatric disorders	> 30% of errors at naming tests and on FAB	KPS of < 70, multifocal lesions	Uncontrolled seizures	Age: not under 18 years old
Rughani, A.I., et al., 2011 (64)	18	6 vs 12	AAA	Inability to cooperate	Cognitive deficits	Outside eloquent areas		
Pereira, L.C.M., et al., 2009 (65)	79	41 vs 38	FA	Inability to cooperate, psychologically instability	Severe Aphasia	KPS of < 70, lesions extending to the thalamus, hypothalamus or brainstem, diameter of lesions larger than 10 cm	Systemic diseases, ASA ≥3	
Gupta, D.K., et al., 2007 (66)	24	16 vs 8	FA	Inability to cooperate, developmental delay	Severe Aphasia	Hemiplegia		Age: not under 12 years old

AAA, asleep-awake-asleep; AC, awake craniotomy; ASA, American Society of Anesthesiologists; BMI, body mass index; FAB, frontal assessment battery; HGG, high grade glioma; LGG, low grade glioma; MMSE, Mini Mental State Examination; MRCMS, Medical Research Council Muscle Strength scale; NA, not available; OCD, Obsessive-compulsive disorder.

#### 3.3.1 Psychological exclusion criteria

Among the psychological exclusion criteria, cooperation inability and psychiatric conditions were the most recurrent, being respectively cited in 10 (22%) and 9 (20%) papers. In particular, direct mention of anxiety disorders was present in 5 (11,1%) articles, whilst 3 (7%) studies described different degrees of confusion and/or disorientation. Emotional disorders such as alexithymia or emotional instability were reported in 5 articles (11,1%). Developmental delay was cited in 2 (4,4%) papers. Only 2 (4,4%) studies reported quantitative measures to diagnose depression or anxiety levels.

#### 3.3.2 Neuropsychological exclusion criteria

The most widely accepted cognitive exclusion parameters were severe aphasia, reported in 24 (53,3%) studies, and global cognitive impairment, reported in 10 (22,2%) studies. A few articles mentioned dysphasia (5 papers, 11,1%) and impairments affecting cognitive functions other than language (2 papers, 4,4%). Seven (15,5%) articles reported a specific percentage of errors at cognitive tests: 3 (6,7%) studies concerning object naming, 3 concerning global cognitive assessment (MMSE) and 1 (2,2%) addressing frontal assessment.

#### 3.3.3 Neurological/neurosurgical exclusion criteria

Serious strength or cognitive deficits represented the most cited neurological exclusion criteria, being reported in 13 (28,8%) studies: a few of them used a neurological scale to quantify the deficit. Recurrent uncontrolled seizures were reported as exclusion criteria in 7 (15%) studies. Three papers (6,7%) indicated that patients were not considered suitable for AS in the case of previous cerebral surgery. Intracranial hypertension and dimensions of the tumor were considered exclusion criteria in 2 studies (4,4%). Finally, both the supposed aggressive histology (HGG) and multifocal lesions were reported as exclusion criteria in 1 (2,2%) paper.

#### 3.3.4 Anesthesiologic exclusion criteria

The most important exclusion criteria according to the anesthesiologic perspective were respiratory and cardiovascular diseases that were reported in 10 (22%) and 7 (16%) papers. Obesity was cited in 8 (18%) studies. Only 2 (4%) studies directly mentioned the American Society of Anesthesiologists (ASA) scale. Reduction in mouth opening and severe gastroesophageal reflux were cited in a few articles. Finally, the presence of diabetes mellitus was considered in 1 paper (2,2%).

#### 3.3.5 Other parameters

Age is one of the most discussed elements, being reported in 10 articles (22,2%), whilst patient's refusal was specifically addressed in 1 paper (2,2%).

## 4. Discussion

Resective surgery often represents the first and main treatment option for patients with gliomas. It is based on the subtle balance neurosurgeons experience every day while striving to remove an infiltrating, malignant tumor in the brain without violating patients' functional status and brain connections. In 2012, De Witt Hamer et al. published a meta-analysis of the literature evaluating the impact of intraoperative stimulation mapping (ISM) on brain glioma surgery (67). The authors found that ISM was associated with improved EOR and reduced risks of definitive neurological deficits, stating the universal adoption of ISM should be considered as the standard of care for glioma surgery, mostly in eloquent areas. In a recent score-matched analysis of an international and multicenter cohort study, AS related to longer OS and PFS as well as fewer definitive neurological deficits compared to asleep craniotomies for patients suffering from glioblastoma (68). To date, superior cognitive functions, such as language and executive functions, as well as dexterity, ideo-motor praxis and visuospatial pathways can be monitored through ISM only performing AS.

AS has changed and evolved over the years in regards to both its technical and conceptual aspects. Nonetheless, the indication and eligibility of glioma patients for this technique are not well established. Moreover, they often relate to center tradition and preferences as well as to surgeon experience and beliefs. This scoping review aims at shedding light on this intriguing topic, potentially helping young neurosurgeons and centers with less experience with the technique to better understand the selection process for addressing patients with AS.

### 4.1 Indication to AS: Tested functions

#### 4.1.1 Testing of language

As shown in **Figure 3**, language is the most frequent superior function being tested in the surgical series included in this study as well as the earliest one. A recent meta-analysis demonstrated that awake craniotomy with electrical stimulation is associated with better long-term language outcomes, as well as higher chances of GTR and shorter in-ward stay (69).

Along with the paradigm shift from language as a function purely related to Broca and Wernicke areas to the hodological view of language as a result of parallel and large-scale distributed network interactions (70), ISM techniques during awake language monitoring have been subjected to important changes in recent years.

For example, the seminal work of Haglund and colleagues in 1994 (71) was based purely on cortical stimulation mapping to identify essential language sites, as stated by the authors. Nowadays, awake language monitoring involves extensive stimulation of both cortical areas and subcortical white matter tracts implied in language comprehension and production (72, 73). Particularly, articulatory disturbances could be elicited by stimulating the ventral premotor cortex, while stimulation of the pars orbitalis could cause semantic paraphasias (74). The latter might be also elicited when the inferior fronto-occipital fasciculus (IFOF) is stimulated (74). Phonemic paraphasias are related to superior longitudinal fasciculus (SLF) and arcuate fasciculus (AF) stimulation (74).

In this light, the non-dominant hemisphere is increasingly recognized to have a potential role in language functions. As per this review, the surgical series encompassing language monitoring of either the dominant and non-dominant hemispheres began to be reported in 2000 (75, 76) and those assessing language skills solely in the non-dominant hemisphere were reported starting from 2017 (77–79). Nonetheless, in the last decade monitoring of language functions has been applied predominantly to the dominant hemisphere: 90 (84%) studies included patients with gliomas in the left hemisphere, 12 (11%) studies included patients with gliomas located in the left or the right hemisphere, and only 3 (2,8%) studies included patients with gliomas in the right hemisphere.

Concerning intraoperative tasks, language monitoring was addressed mainly *via* the object (104 studies) and verb (21 studies) naming tests. Semantic association was tested in 20 (11,3%) articles. The non-verbal semantic association was frequently assessed through the pyramids palm and trees test (PPTT), which was used by Corrivetti et al. to find that errors in the semantic association domain were associated with pre-SMA stimulation (80), as well as by Prat-Acin et al. to show that dorsolateral prefrontal cortex stimulation is involved in the disruption of semantic processing (73). Stimulation of the pre-SMA should also be conducted during spontaneous speech because of the role of pre-SMA and FAT in spontaneous speech initiation and verbal fluency (81). The verbal semantic association were largely evaluated by the DO 80 picture naming task, which was invalidated by stimulation of the dorsolateral prefrontal cortex, pars triangularis and pars opercularis to a cortical level, and stimulation of the IFOF to a subcortical level (77, 82). De Witte et al. also suggested the employment of a lexical-semantic processing test to intraoperatively assess the verbal semantic association (83). It is available in multiple languages and might be employed to monitor verbal semantic association.

Writing and reading functions were respectively investigated in 2 (1%) and 28 (16%) articles. Lubrano et al. found the frontal lobe, particularly dominant F2 and F3, to be involved in these functions (84). Intriguing, stimulation of F2 determined irregular handwriting and words impossible to decipher, with the main writing disturbance being represented by orthographic errors. On the other hand, stimulation of F3 determined word or letter substitutions, paragraphia, and/or writing arrest. Reading was also showed to be disrupted by stimulation of the angular and supramarginal gyrus (83).

#### 4.1.2 Testing of sensorimotor functions

Gliomas in the precentral gyrus have been considered unresectable for years. The introduction of ISM drastically changed this perspective as proved by the increasing number of case series in the literature that focused on this topic (**Figure 3**). The surgical technique of ISM for the resection of gliomas involving motor areas and pathways might be essentially reconnected to two main strategies: cortical and subcortical mapping with continuous motor evoked potential (MEP) monitoring, with or without awake craniotomy. While patients with gliomas in motor areas of the dominant hemisphere are likely to be operated on in awake settings (for example to monitor and preserve language and/or visuospatial functions as well), AS for gliomas in motor areas and pathways of the non-dominant hemisphere is often related to centers and neurosurgeons' preference and expertise. The Well diagram in **Figure 4** demonstrated that only 20 studies, the 24% of the surgical series testing sensorimotor functions, monitored only the motor pathway during AS.

Concerning the mere motor monitoring, Saito et al. stated that the most useful advantage of combining awake surgery with continuous MEP monitoring is that the surgeon can monitor the motor function by directly observing patient intraoperative voluntary movements (IVMs) while comparing MEP changes that could be sometimes inaccurate as in case of brain shift (85). Nevertheless, a recent meta-analysis concluded that gliomas located near or in the motor areas of the brain can be safely carried out with either asleep and awake protocols, without differences in terms of EOR and definitive postoperative deficits (86).

In a recent experience on the praxis pathway stimulation, ideo-motor apraxia seemed not to be directly affected by M1 and pyramidal tract stimulations, but rather by direct electrical stimulation of the ventrolateral premotor cortex and supramarginal gyrus as well as of the subcortical frontoparietal white matter (87). As a consequence, the authors favored the use of awake motor mapping through an intraoperative motor task for tumors located in the praxis network in patients with no preoperative deficits, while suggesting an asleep motor mapping for patients with lesions near or involving the central sulcus, or patients with preoperative deficits or history of previous treatments (88).

While monitoring of M1 and pyramidal tract in asleep patients is reliably addressed by high frequency (HF) stimulation at the cortical and subcortical levels, stimulation of praxis circuits requires the use of low frequency (LF) technique during hand movement performance both at the cortical and subcortical level; hence, mapping of praxis tracts mandates AS.

Since buccofacial apraxia and limb apraxia seem to be related to different pathways (89), Morrison et al. identified the area of speech apraxia by LF DES of the lower pre- and postcentral gyri that were identified as areas of tongue movement by preoperative fMRI (90).

Three major studies mainly focused on multilanguage patients, demonstrating that the sites for each language were different and separate (21–23). In all three studies, the surgical time seemed not to be affected by intraoperative mapping of cortical and subcortical sites of the different languages. Sellier et al. reported the results obtained from 84 foreign patients and 18 different intraoperatively tested languages; the authors showed that the presence of the translator in the theatre allowed all the intraoperative tasks to be completed, with the rate of postoperative deficits being not affected by the inability of patients to communicate with the team. In the meantime, the EOR appeared to be inferior when dealing with foreign or multilanguage patients. Finally, ReFaey et al. highlighted that multilingual patients received higher DES current without influencing the rate of intraoperative seizure rates.

#### 4.1.3 Testing of visuospatial pathway

As stated by Bertani et al., visuospatial mapping during awake surgery is usually performed in patients with lesions located in the parietal lobe and often is intermingled with language mapping (6). The test which resulted more frequently exploited in this review is the line bisection task. It is usually performed at the cortical and subcortical levels to avoid neglect and visual field deficits (6, 91).

#### 4.1.4 Testing of executive functions and emotions

We merged the testing of executive functions and theory of mind/emotions in this paragraph because of the emerging interest these functions gained in the last decade.

Inhibition plays a fundamental role in decision making (one of the main executive functions) and lends itself to be tested during AS through the Stroop test. As suggested by the reviewed surgical series, testing of executive functions might be particularly useful when dealing with tumors of the anterior cingulate cortex (92).

The theory of mind is described as the process of inferring others' mental states, which relates to emotions, thoughts and feelings (78). It can be intraoperatively tested *via* the "Reading the Mind in the Eyes" (RME) test (93). The areas involved in social cognition and the Theory of Mind were identified variously among the awake series of this review. Yordanova et al. identified two clusters of responsive stimulations, one in the dorsolateral prefrontal cortex and the other in the right inferior frontal gyrus (IFG) (78). Nakajima et al. related the premotor and posterior parts of the prefrontal cortices to various kinds of basic emotions, demonstrating that the preservation of a positive site is associated with basic emotion function preservation even in cases of transient decline after surgery (94).

Since dysexecutive syndromes are characterized by behavioral and cognitive symptoms that contribute to loss of autonomy (95), awake monitoring of executive functions might theoretically reduce their occurrence, improving patients' quality of life (35, 73). Nonetheless, the results of this review pointed out the still restricted evidence about the oncological and functional impact of higher executive functions' intraoperative monitoring. A thorough cognitive assessment performed in conjunction with language testing should be a necessary step in the global evaluation of glioma patients, both before and after surgery, to investigate the added value of testing higher cognitive functions intraoperatively (63).

#### 4.1.5 Indication to AS – Final commentary

Indications for AS changed over the years as well as its technical and conceptual aspects. The preservation of language is the most frequent and widely recognized indication to awake patients. The increasing application of AS and the unceasing study of brain connections made it possible to map different language networks as proved by the growing surgical series in this review that tested domains different from those classically related to speech production and naming. Hand dexterity and praxis are essential functions that might heavily impact patient quality of life. Their preservation represents the main indication to awake patients with gliomas affecting sensorimotor areas. The visuospatial pathway should be tested every time neurosurgeons suspected the patient might be at risk of developing neglect or hemianopia since they could increase morbidity. The line bisection task proved to be reliable and reproducible for patients with gliomas near optic radiations. Finally, executive functions and Theory of Mind could be addressed by simple intraoperative tests, such as the Stroop and RME tests. They are usually employed when dealing with lesions affecting the anterior cingulate and the dorsolateral, premotor and prefrontal cortices.

In accordance with the results of this scoping review, a summary of the implemented intraoperative tasks and tested function being assessed in relation to specific brain regions is available in **Table 2**.

**Table 2:** Implemented intraoperative tasks and tested function being assessed in relation to specific brain regions.

Lobe (Regions)	Function	Test
***Frontal Lobe*** ** **		
*Rolandic and Peri-Rolandic Area*	Praxis	**Hand and Tongue Fine Movement tasks**
* *		** **
*Anterior Cingulate Cortices*	Inhibition	**Stroop Test**
* *		** **
*Right IFG and dlPFC*	Theory of Mind	**RME test**
* *		** **
*Left IFG*	Language	**Spontaneous Speech**
* *		**Counting**
* *		**Picture Naming test**
* *		**Verb Generation test**
* *		** **
*Left Inferior and Middle Frontal Gyri*	Reading	**Reading task**
* *	Writing	**Writing task**
* *		** **
*pre-SMA*	Non-Verbal Semantic Association	**PPTT**
* *		** **
* *	Speech Initiation and Verbal Fluency	**Spontaneous Speech**
* *		** **
*dorsolateral prefrontal cortices (dlPFC) and Left IFG*	Verbal Semantic Association	**Picture Naming test**
		**DO 80**
* *		**Lexical-Semantic Processing test**
***Parietal Lobe*** ** **		
*Left SMG and AG*	Language	**Spontaneous Speech**
* *		**Picture Naming test**
* *		**Verb Generation test**
* *		** **
* *	Reading	**Reading task**
* *		** **
*Inferior Parietal Lobule*	Visuospatial Pathway	**Line Bisection task**
***Temporal Lobe*** ** **		
*Left Superior and Middle (Posterior) Temporal Gyri*	Language	**Spontaneous Speech**
* *		**Picture Naming test**
* *		**Verb Generation test**
*Inferior Parietal Lobule*	Visuospatial Pathway	**Line Bisection task**
***Insula*** ** **		
*Left Insula*	Language	**Spontaneous Speech**
* *		**Picture Naming test**
* *		**Verb Generation test**
***Occipital Lobe*** ** **		
	Visuospatial Pathway	**Line Bisection task**

AG, angular gyrus; dlPFC, dorsolateral prefrontal cortex; IFG, inferior frontal gyrus; SMG, supramarginal gyrus.

### 4.2 Eligibility criteria for AS – Exclusion criteria

#### 4.2.1 Psychological exclusion criteria

Cooperation and active participation of the patient are the most important factors for a successful procedure (29). Therefore, one of the most cited exclusion criteria is the impossibility for the patient to positively cooperate with the surgical team: during AS, non-compliant behavior may cause significant and severe complications which may at least determine the abortion of the awake phase. Inability to cooperate may be due to several reasons, from disorganized or apathetic behaviors to cognitive deficits or personality structure issues (6, 34). Another possible cause of uncooperative behavior is the lack of sufficient confidence in the medical team, an aspect highlighted by Huguet et al. (32) and by Hejrati et al. (53), which pointed out the importance of sensible management of the patient and relatives. In this regard, the level of motivation is another considered psychological aspect (32); it should not be underestimated the attention dedicated by clinicians to this issue and the need for thorough and comprehensive explanations to the patient and relatives. In the authors' experience, a strong bond between the surgical team and the patient and its family increases motivation and facilitates positive cooperation with AS, while helping the patient to cope with anxiety and fear of surgery, especially in the intraoperative awake scenario.

Another well-established exclusion criterion is the presence of psychiatric diseases such as psychotic or personality disorders, obsessive-compulsive disorder and depression (32, 46, 52, 96). These elements can be assessed through the anamnestic recollection but, in case of doubts, specific questionnaires or scales can also be administered. From this review, Santini and colleagues (96) used the following scales to diagnose psychiatric symptoms and, in case of clinically significant scores, they excluded the patient: the Beck Depression Inventory (97) for depressive symptoms and the State-Trait Anxiety Inventory (98) for anxiety levels. Goebel et al. (47) suggested using the Hospital Anxiety and Depression Scale (HADS) (99).

Anxiety is the most frequently assessed psychopathological disorder, including its different sub-types such as panic attacks and phobias (31, 35, 37, 40, 47, 52, 56, 58, 61). A careful examination of these aspects can predict the ability to control anxiety and fear related to AS. Undoubtedly, some levels of anxiety or mood deflection are frequently found in hospitalized patients; it is fundamental to understand whether it is possible managing them through specific psychological work before surgery, or whether they are too severe to enable positive cooperation before and during AS.

Depressive disorders can affect cooperation during surgery since depressive symptoms may reduce pain tolerance levels (53, 96).

Emotional lability and alexithymia were also considered as possible exclusion criteria (32, 38, 48, 57, 65) as well as confusion, disorientation and developmental delay, due to the consequent inability to understand the surgical procedure and cooperate during it (25, 35, 37, 57, 66).

#### 4.2.2 Neuropsychological exclusion criteria

Nowadays, a neuropsychological evaluation is considered mandatory for AS procedures, as it allows clinicians to detect specific cognitive alterations for each patient to verify the presence of a pre-operative cognitive deficit that would preclude correct tailoring and execution of intraoperative tests (6, 32, 100). Most of the included works provided only a general description of the neuropsychological parameters to be taken into account during the selection phase of patients for AS and focused mainly on language testing. Severe aphasia is a widely described criterion, though rarely further specified; only a few articles (34, 46, 58, 59, 96) specify a percentage (50%, 25% or 20%) of errors as the cut-off for patient inclusion or exclusion. In the case of a pre-existing preoperative language impairment, the awake team should tailor the intraoperative tasks to the patient needs and abilities, keeping in mind that a severe impairment may be present in isolated linguistic levels while other modalities/levels could be still intact. As reported by De Witte et al., the items the patient is unable to perform correctly in the preoperative assessments should be left out of the set for intraoperative testing to ensure that the errors in the awake setting are due to cortical stimulation and not caused by a pre-existing deficit (83).

Poor global cognitive status was generally reported as an exclusion criterion as it prevents patients from a correct comprehension of the procedures. In some cases (34, 42, 47), an impaired Mini-Mental State Examination (MMSE) (101) was used as an objective parameter to evaluate a global cognitive impairment, although used alone it is not considered an extensive instrument to describe patients' cognitive abilities.

Dysphasia was another reported parameter in several studies (31, 33, 37, 48, 54), due to its implications concerning language production.

Impairments involving cognitive functions other than language, such as neglect or attention, were rarely reported as they do not necessarily represent exclusion parameters for language monitoring.

To maximize the coherence among evaluations and to increase the reliability of patient answers during the mapping phase, a few works (6, 34, 37, 42) recommended that both the preoperative and intraoperative tests should be administered by the same neuropsychologist; patients would also be reassured by the presence of a known person talking with them during AS.

#### 4.2.3 Neurological/neurosurgical exclusion criteria

Concerning patient functional and neurological status, the Karnofsky Performance Status (with a score below 70), the Medical Research Council Muscle Strength scale (score < 2) and the LOVETT scale (score < 3) were often implemented as useful tools on which patient eligibility is based on (30, 35, 36, 51, 59, 61, 65, 96).

Another exclusion criterion was represented by uncontrolled epilepsy, which might increase the intraoperative risk of seizures (27, 37, 40). Previous brain surgery is a less frequently cited exclusion criterion (24, 27, 102) as well as considerations about tumor dimension: Pereira et al. (65) stated excluding patients for AS due to tumor diameter superior to 10 cm, while Garavaglia et al. (61) excluded large lesions provoking midline shift. Finally, another rarely cited parameter was endocranial hypertension (40, 42). In the authors' opinion, endocranial hypertension compromising the patient neurological status constitutes an obvious exclusion criterion. Concerning tumor volume, it should not necessarily represent a negative factor, even if it might be related to longer surgical time thus negatively affecting patient cooperation. In this view, AAA technique might be preferred in case of larger tumors, limiting the awake period duration to that strictly necessary to complete the functional boundaries delimitation.

4.2.4 Anesthesiologic exclusion criteria

Two main disease categories were widely considered as exclusion criteria: respiratory and cardiovascular. Among the first group, difficult airways, obstructive sleep apnea, asthma and severe chronic obstructive pulmonary disease were exclusion criteria due to the difficulties of intubation and sedation they may provoke (6, 26, 36, 37, 40, 42, 45, 49, 61, 62).

Cardiovascular disorders are less detailed, only severe cardiomyopathies and the presence of pacemakers were further specified (6, 26, 30, 36, 37, 40, 42, 60).

Several studies recognized obesity as an exclusion parameter and, in some cases, with a Body Mass Index cut-off of 35 (37, 62). This criterion was adopted in a preventive view to reducing the incidence of aspiration (62) or other complications that would be difficult to manage in the awake setting, such as airway obstruction due to oversedation with an exposed and vulnerable brain, or hypercarbia, a potential stimulator of the cerebral blood flow which might lead to intraoperative bleeding, brain swelling and increased surgical difficulties (61).

Gastroesophageal reflux was considered an exclusion criterion due to possible complications with the positioning of the laryngeal mask (53, 61, 62).

Some clinicians reported using the American Society of Anesthesiologists scale, with an exclusion cut-off score of 3, to maximally objectify the eligibility decision according to an anesthesiologic point of view (30, 65).

#### 4.2.5 Other exclusion criteria

Age was widely considered as a possible exclusion criterion, even if standardization is lacking: lots of works described AS for patients older than 60 (6, 13, 25–27, 34, 38, 41, 42, 45, 46, 49, 60) or than 70 years of age (33, 35, 39, 47, 53, 56, 58, 61, 64–66, 96). In some cases, also elderly people were awakened (29, 31, 39, 40, 48, 52). On the other hand, considering the pediatric population (< 18 years old), several works adopted AS for children and adolescents (6, 24, 25, 29, 32, 33, 39, 43, 48, 50, 52).

In the pediatric population, age may influence the possibility for young patients to sufficiently understand and cooperate. Huguet and colleagues (32) report that AS in children should be carefully evaluated due to higher psychological fragility and increased surgical risks, though if well selected and prepared, children can have a similar awake outcome to that of adult patients.

A few studies reported excluding only very young children (<10-13 years) (30, 43, 66), whilst others restrict the population eligible for AS to adolescents and adults (16-18 years) (25, 55, 60, 96). One of the main concerns regarding AS in children could be the use of intraoperative tasks that had been standardized for the adult population. Collée et al. recently reported the case of a 12-year-old child undergoing AS with the use of standardized language tests for children. According to the authors, language tests for children allow a careful assessment of language in younger people, while excluding the possibility of a concomitant developmental language disorder (103).

Concerning histology, only one paper declares to exclude patients if HGG is suspected (44), though without further explanations. A recent meta-analysis by Zhang et al. (104), which investigated outcomes of AS for glioblastomas (GBMs), demonstrated the viability of AS for GBM resection in or near eloquent areas. Particularly, the use of AS was associated with a low rate of persistent postoperative neurological deficits (1,9%) while achieving an acceptable rate of GTR (74,7%).

#### 4.2.6 Exclusion criteria – Final commentary

The findings of this scoping review pointed out that the eligibility criteria for AS are still poorly standardized.

The inability of the patient to cooperate represented a shared exclusion criterion among the included surgical series. Likewise, the presence of a severe language deficit before surgery was generally accepted as an exclusion criterion for AS. Nonetheless, very few papers in our sample mentioned specific deficient language levels or exact cut-off values for grading the preoperative language impairment that were used during the patient selection process for AS.

Similarly, many papers deemed psychological disorders (i.e. anxiety) and other medical conditions (i.e. cardiovascular diseases, obesity, etc.) as exclusion criteria, without specifying any objective parameters to exclude patients from AS.

Concerning patient age and tumor histological features, this review included patients with an age range from 9 to 90 years who were affected by different tumor types and grades of brain gliomas.

## 5. Limits

The main limitation of the paper is that relevant pieces of information regarding age, gender, histology and surgery protocol lack in several of the included studies, thus leading to a significant loss of information for our work. Some of these elements have been investigated only in a few articles, preventing us from having a wider sample of information to describe and compare.

## 6. Conclusions

The results of this review suggest that the indication and eligibility of patients for AS are still not fully standardized. The main indication to awaken patients with gliomas in eloquent areas is represented by language monitoring, even if tasks and stimulation techniques changed over the years. The sensorimotor pathway is the second most frequent indication of AS. Even if the mere monitoring of the pyramidal tract might be addressed in asleep settings, growing attention to dexterity and praxis emerged in the last decade. In the meantime, this review demonstrated an increasing interest in functions different from those of language and sensorimotor pathways such as executive functions, social cognition and emotions that might be addressed when dealing with lesions of the anterior cingulate and the dorsolateral, premotor and prefrontal cortices.

Inability to cooperate, severe aphasia, psychological disorder, such as pathological anxiety and depression, or medical conditions, such as severe respiratory and cardiovascular diseases, or obesity, are shared exclusion conditions that however are not identified through standard cut-offs. Future efforts should be focused on the quantitative evaluation of these variables, in order to minimize uncertainties in patient selection and safely expand the pool of candidates for awake surgery.

**Figure 5:**
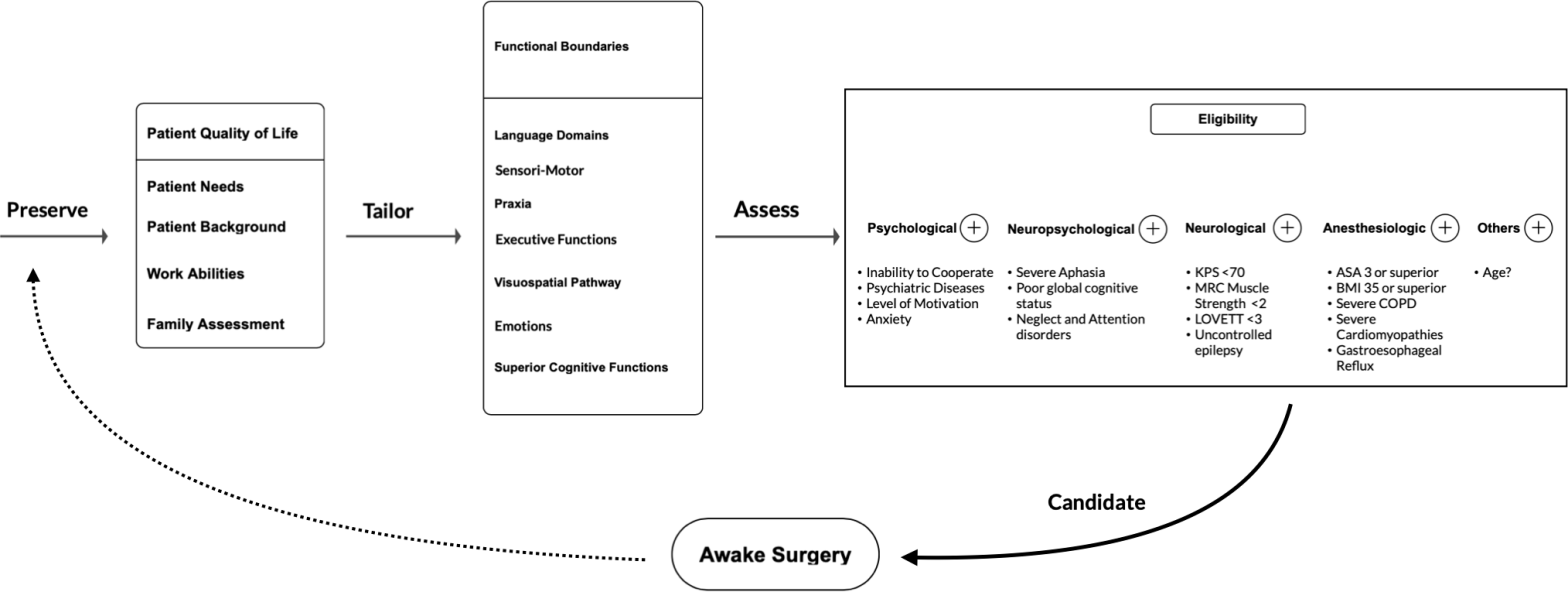
Flow-chart of the proposed patient selection process for indication and eligibility to AS suggested by the authors on the basis of this scoping review results.

Given the broad spectrum of functions that might be safely and effectively investigated and preserved *via* awake mapping and monitoring, in line with the growing body of the recent literature, this technique should no longer be confined to lesions located in areas classically considered eloquent and/or to the dominant hemisphere. Neurosurgeons and their teams should tailor intraoperative testing on individual patient needs, abilities and brain connectivity, as well as on tumor location and features. Whenever the aforementioned exclusion criteria are not fulfilled, AS should be strongly considered for glioma patients (**Figure 5**).

## Authors contributions

GF: study design and methodology, data and statistical analysis, figures and graphs, wrote the draft, finally reviewed the manuscript. GA-F: data collection, data analysis, tables, wrote the draft. AF and LT: data collection and analysis, tables. CB, VC, MV, FC, MC, MP, SB: data analysis and finally reviewed the manuscript. ML and GB: study design and methodology, data analysis, revised the draft and finally reviewed the manuscript. All authors contributed to the article and approved the submitted version.

## Data availability statement

The original contributions presented in the study are included in the article/supplementary material. Further inquiries can be directed to the corresponding author.

## Funding

This study , as well as its dissemination, was supported by "Associazione Amici della Clinica Neurochirurgica".

## Conflict of interest

The authors declare that the research was conducted in the absence of any commercial or financial relationships that could be construed as a potential conflict of interest.

## Publisher’s note

All claims expressed in this article are solely those of the authors and do not necessarily represent those of their affiliated organizations, or those of the publisher, the editors and the reviewers. Any product that may be evaluated in this article, or claim that may be made by its manufacturer, is not guaranteed or endorsed by the publisher.
